# Supplementary material for: Multiple Myeloma-Derived Extracellular Vesicles Induce Osteoclastogenesis through the Activation of the XBP1/IRE1α Axis
Source: Cancers (Basel). 2020 Aug 4;12(8):2167. doi: 10.3390/cancers12082167 (PMC7465175; doi:10.3390/cancers12082167)
Supplement: Supplementary file 1 [file cancers-12-02167-s001.zip › cancers-889371 -Supplementary.pdf]

Supplementary Materials: Multiple Myeloma-Derived Extracellular Vesicles Induce Osteoclastogenesis through the Activation of the XBP1/IRE1α Axis

Lavinia Raimondi, Angela De Luca, Simona Fontana, Nicola Amodio, Viviana Costa, Valeria Carina, Daniele Bellavia, Stefania Raimondo, Sergio Siragusa, Francesca Monteleone, Riccardo Alessandro, Milena Fini and Gianluca Giavaresi

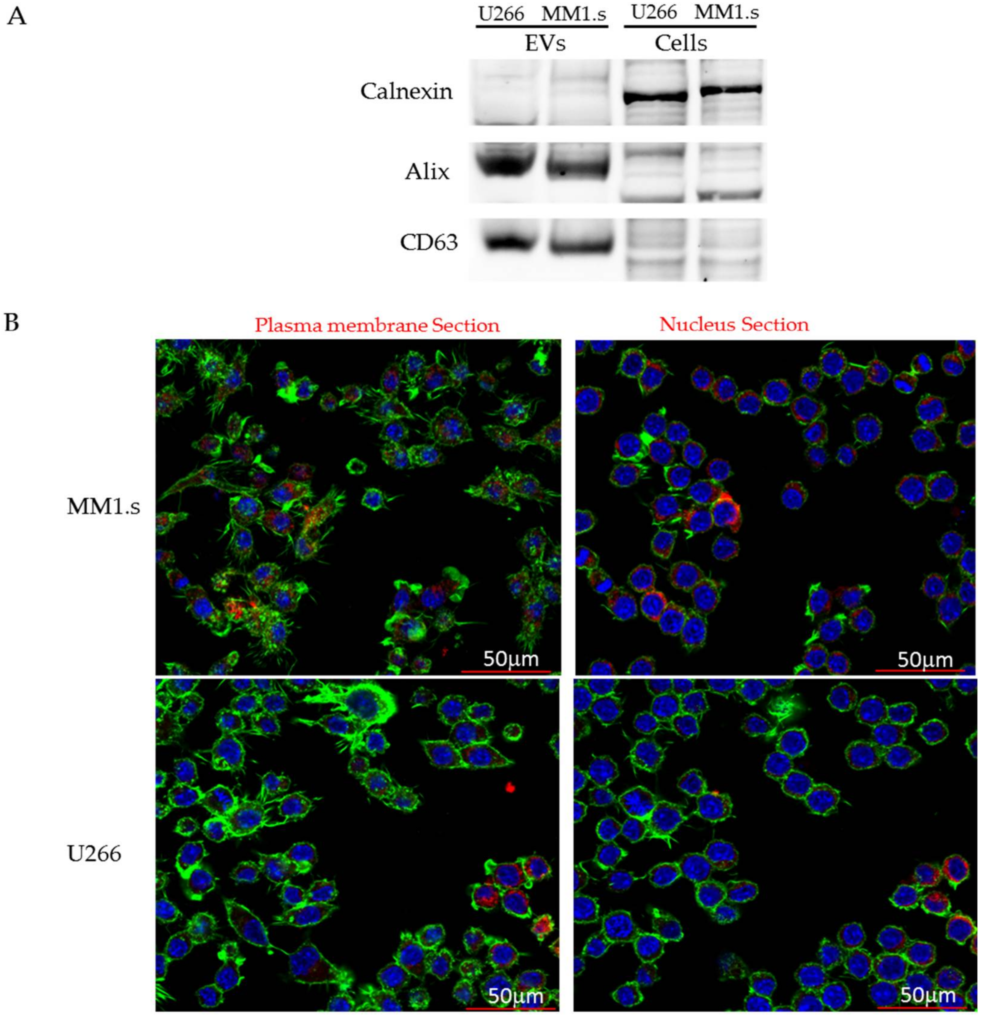

Figure S1. A. Western blotting analysis of Alix, CD63 and Calnexin in U266−, MM1.s-EVs and cellular lysates. B. Uptake of MM-Evs by Raw264.7 cells; analysis at confocal microscopy of Raw264.7 cells treated for 6 hours with 25 µg/ml of MM1.s- and U266-EVs. Raw264.7 cells were stained with Actin green (green), nuclear counterstaining was performed using Hoescht (blue) and exosomes were labelled with PKH26 (red). Scale bar = 50 µm.

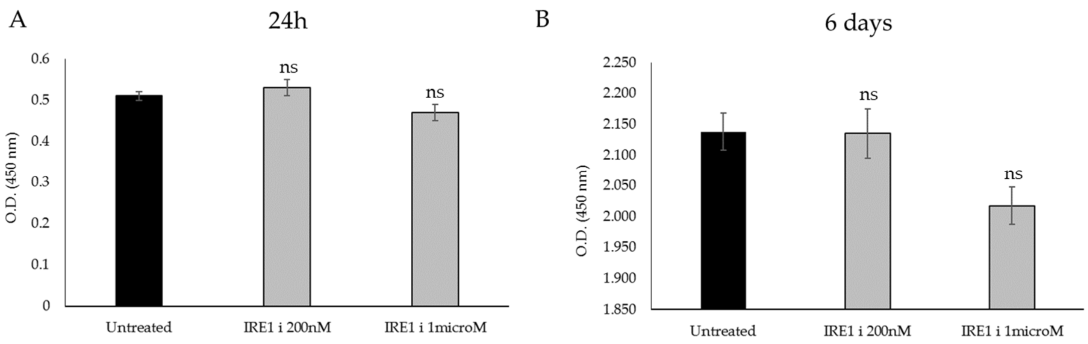

Figure S2. Cell viability assay was evaluated through WST-1 assay of Raw264.7 cells untreated or Table 200. nm or 1microM of IRE1a inhibitor, at 24 hours (A) and 6 days (B) of culture.

	Area values for loading band: Tubulin
RL	13.581
Untr	12.089
Evs MM1.s 3h	11.801
6h	11.006
24h	11.779
DTT	12.810
Evs U266 3h	13.352
6h	14.111
24h	13.434

	Area values for loading band: TOT IRE1a
RL	3.602
Untr	4.333
Evs MM1.s 3h	3.152
6h	4.132
24h	3.519
DTT	5.070
Evs U266 3h	4.586
6h	5.143
24h	5.372

	Area values for loading band: p-IRE1a
RL	2.647
Untr	552
Evs MM1.s 3h	780
6h	2.558
24h	3.413
DTT	5.380
Evs U266 3h	749
6h	1.292
24h	6.718

Densitogram analysis Western Blotting Figure 2A

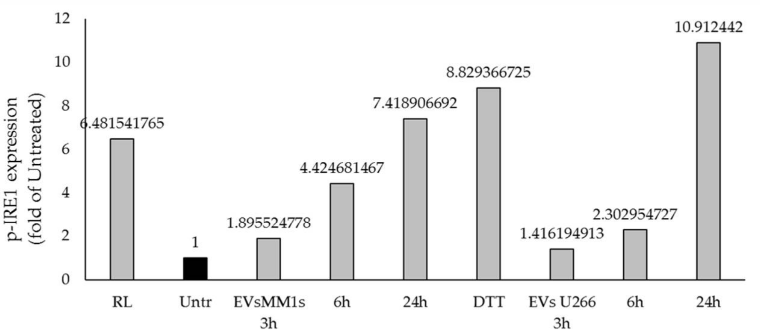

Area values for loading bands from western blot **Figure 2A**

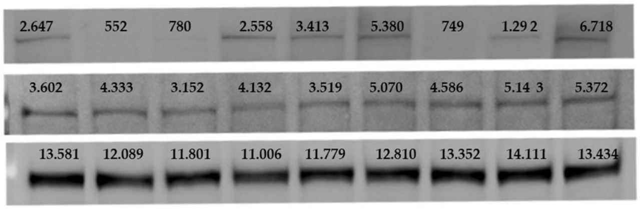

Western Blot Figure 2A

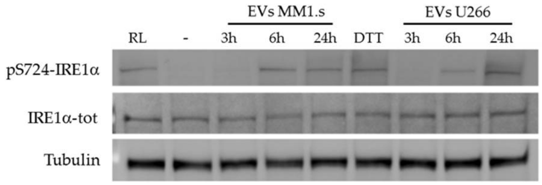

IRE1 alpha antibody (**p-Ser724**) [NB100-2323]

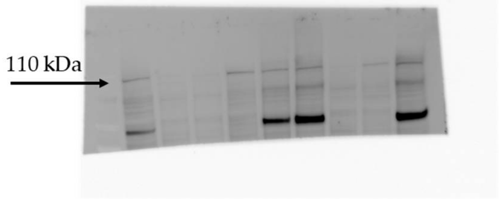

Tubulin

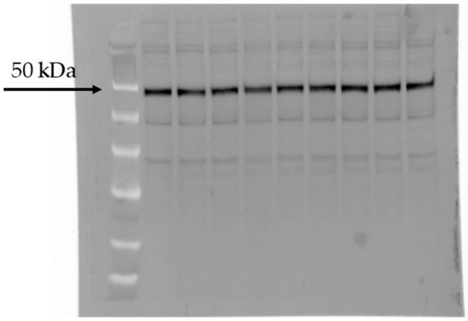

IRE1 alpha antibody (**Tot**) [NBP2-50067]

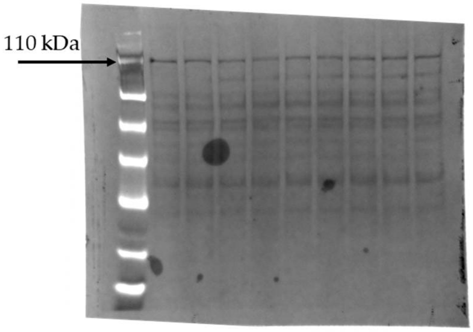

Figure S3. Whole Western Blot for Figure 2A.

Area values for loading bands from western blot **Figure 3A**

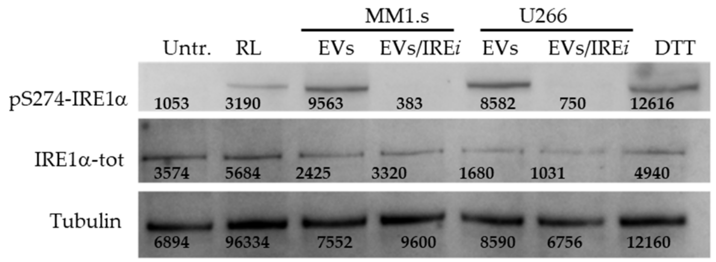

Densitogram analysis Western Blotting Figure 3A

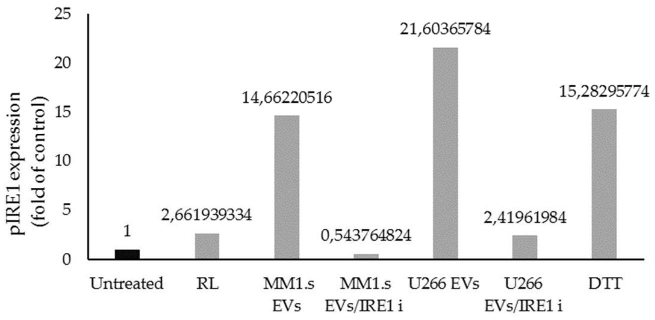

Area values for loading band: Tubulin		Area values for loading band: TOT IRE1 a	Area values for loading band P-IRE1 a
6.894		3.574	1.053
9.634		5.684	3.190
7.552		2.425	9.563
9.600		3.329	383
8.590		1.680	8.582
6.756		1.031	750
12.160		4.942	12.616

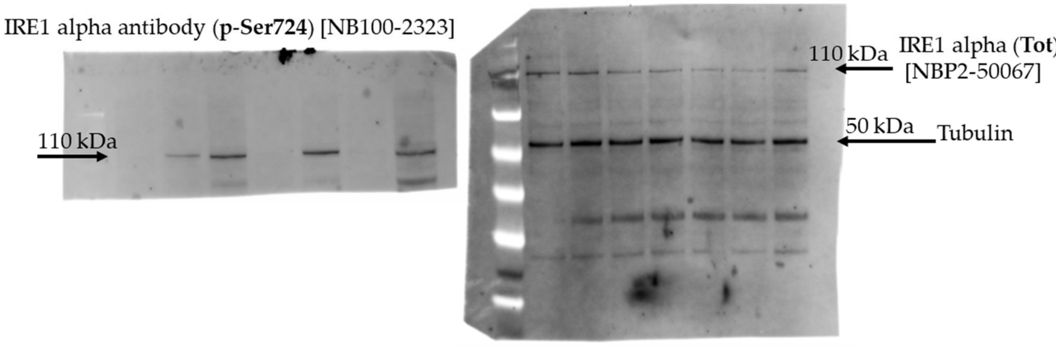

Figure S4. Whole Western Blot for Figure 3A.

© 2020 by the authors. Licensee MDPI, Basel, Switzerland. This article is an open access article distributed under the terms and conditions of the Creative Commons Attribution (CC BY) license (http://creativecommons.org/licenses/by/4.0/).
